# Harnessing the Potential of Quinoa: Nutritional Profiling, Bioactive Components, and Implications for Health Promotion

**DOI:** 10.3390/antiox13070829

**Published:** 2024-07-10

**Authors:** Xiaomin Xi, Guanghe Fan, Huimin Xue, Shuai Peng, Weidong Huang, Jicheng Zhan

**Affiliations:** College of Food Science and Nutritional Engineering, China Agricultural University, Beijing 100083, China; xxm1113@cau.edu.cn (X.X.); fanguanghe@cau.edu.cn (G.F.); 2018319010217@cau.edu.cn (H.X.); shuaipeng1113@163.com (S.P.); weidonghuang@cau.edu.cn (W.H.)

**Keywords:** quinoa, pseudocereal, nutrients, bioactive components, health benefits

## Abstract

Quinoa, a globally cultivated “golden grain” belonging to *Chenopodium* in the *Amaranthaceae* family, is recognized for being gluten-free, with a balanced amino acid profile and multiple bioactive components, including peptides, polysaccharides, polyphenols, and saponins. The bioactive compounds extracted from quinoa offer multifaceted health benefits, including antioxidative, anti-inflammatory, antimicrobial, cardiovascular disease (CVD) improvement, gut microbiota regulation, and anti-cancer effects. This review aims to intricately outline quinoa’s nutritional value, functional components, and physiological benefits. Importantly, we comprehensively provide conclusions on the effects and mechanisms of these quinoa-derived bioactive components on multiple cancer types, revealing the potential of quinoa seeds as promising and effective anti-cancer agents. Furthermore, the health-promoting role of quinoa in modulating gut microbiota, maintaining gut homeostasis, and protecting intestinal integrity was specifically emphasized. Finally, we provided a forward-looking description of the opportunities and challenges for the future exploration of quinoa. However, in-depth studies of molecular targets and clinical trials are warranted to fully understand the bioavailability and therapeutic application of quinoa-derived compounds, especially in cancer treatment and gut microbiota regulation. This review sheds light on the prospect of developing dietary quinoa into functional foods or drugs to prevent and manage human diseases.

## 1. Introduction

Quinoa (*Chenopodium quinoa* Will.), an ancient grain with a long history, has garnered great attention due to its nutritional and health-promoting properties, making it an excellent choice for individuals with coeliac disease and gluten intolerance. Originating from the Andean region of South America, quinoa has been cultivated and consumed for over 5000 years, being revered as the “golden grain” and “mother grain” [1]. The ability to tolerate drought and cold conditions makes it thrive in high-altitude areas and poor soils. Therefore, quinoa cultivation was present in more than 120 countries by 2018, and almost 110 countries have started to test and produce quinoa in the last 30 years, including South America, North America, Asia, Europe, and other regions [2].

As an ancient grain, quinoa showcases a multitude of varieties and diversities classified based on the color, size, and texture of the grains. The most common classification of quinoa is based on color, which includes white, red, and black [3,4]. Quinoa is the sole pseudocereal recognized by the Food and Agriculture Organization (FAO) as a single crop capable of providing all essential nutrients required by humans and ranks seventh on the list of “Top Ten Healthy Nutritious Foods” globally [5]. Overall, the dietary value of quinoa is higher than other grains such as rice, wheat, and corn, with values of 73%, 56%, 49%, and 36%, respectively, approaching that of beef (74%). Quinoa stands out as a superior plant-based protein source, providing nine essential amino acids, notably lysine (4.8 g/100 g protein), threonine (3.7 g/100 g protein), and methionine (1.9 g/100 g protein), which are typically deficient in conventional grains [6,7]. Furthermore, quinoa is abundant in dietary fiber, which is beneficial for intestinal motility and digestive health, particularly in promoting gut homeostasis [1]. Quinoa also serves as a rich mineral source, containing considerable amounts of magnesium, zinc, iron, calcium, potassium, and other essential minerals, with approximately four times higher calcium and iron content compared to other crops [8]. Furthermore, quinoa contains diverse secondary metabolites such as polyphenolic compounds, polysaccharides, saponins, and other bioactive compounds, making quinoa a valuable food source for both humans and animals [9].

As a highly nutritious and gluten-free pseudocereal, quinoa has gained more and more attention due to its high-quality protein, palatable taste, and balanced nutritional profile [10]. To recognize quinoa’s nutritional value, the FAO designated 2013 as the “International Year of Quinoa” and listed it among the top 10 most nourishing foods globally [11]. This review aims to provide an in-depth analysis of quinoa’s nutritional composition and multiple bioactive effects on human health. The remarkable properties and health-promoting benefits of quinoa make it a compelling candidate for inclusion in diverse diets, offering a sustainable and nutritious solution for addressing global nutritional challenges. Without specific emphasis, “quinoa” in this review refers to “quinoa seeds”.

## 2. Nutritional Profile and Functional Components of Quinoa Seeds

### 2.1. Protein and Bioactive Peptides

The protein content of quinoa ranges from 12% to 22%, surpassing that of rice at 7.5%, barley at 11%, and corn at 13.4%. The quality of dietary protein is determined by the ratio of essential amino acids that cannot be synthesized by the human body and must be obtained from diet. The proteins in grains can be categorized into four types: albumins, globulins, prolamins, and glutelins. Unlike other cereal crops that primarily store prolamin proteins, quinoa contains very little prolamin protein (0.5–7%) and glutelin protein (18%). Instead, the main storage protein of quinoa is globulin (37%) and albumin (35%), stabilized by disulfide bonds [6,12]. The low concentration of prolamin in quinoa makes it a suitable alternative for individuals with celiac disease and an attractive option for gluten-free products. The main component of quinoa globulin is 11S globulin, also known as globular globulin. It has a hexameric structure and is composed of six pairs of small, basic polypeptides and larger, acidic polypeptides with molecular masses of 22–23 and 32–39 kDa, respectively [13]. 11S globulin has herein become the reference source for FAO amino acids leucine, isoleucine, phenylalanine, and tyrosine [1]. The secondary structure of quinoa albumin consists of 4% α-helices, 50% β-sheets, and 46% aperiodic structure [14]. Compared to globulins, quinoa albumin is characterized by high levels of cysteine, arginine, histidine, and lysine. Quinoa protein fulfills over 180% of the histidine requirement estimated by the FAO/WHO/UNU, as well as 274% for isoleucine, 338% for lysine, 212% for methionine+cysteine, 320% for phenylalanine+tyrosine, 331% for threonine, 228% for tryptophan, and 323% for valine [15,16]. The lysine content in quinoa is twice as high as in wheat or corn [17]. Therefore, quinoa serves as a high-quality plant-based protein with a well-balanced amino acids profile, addressing deficiencies in lysine and histidine, particularly beneficial for pregnant women and infants. Studies have shown that quinoa protein is highly digestible in animals (about 95.3%), compared to 101.5% for casein; raw quinoa protein is absorbed at a rate of 91.6%, and heat treatment, such as cooking, further increases protein absorption capacity up to 95.3%, making quinoa a suitable food option for individuals with dyspepsia [18]. Due to its high digestibility and superior amino acid profile, the nutritional value of quinoa is comparable to that of beef and far surpasses rice, wheat, and corn [16].

Bioactive peptides (BAPs) are short protein fragments with biological activity derived from specific regions of proteins through enzymatic hydrolysis or proteolytic processes, exhibiting various physiological effects in living organisms [19]. Recent investigations have focused on isolating and characterizing bioactive hydrolysates and BAPs obtained from quinoa proteins with advanced techniques and functional trials [7,20]. The BAPs and protein hydrolysates derived from quinoa have displayed numerous bioactive effects, including antioxidant, anti-inflammatory, and anti-cancer effects, especially in terms of hypoglycemic, hypotensive, and hypolipidemic activities, which have been widely studied in vitro and in vivo [21,22,23]. Table 1 summarizes several BAPs derived from quinoa protein, along with their sources and analytical methods.

### 2.2. Carbohydrates and Dietary Fiber

Carbohydrates represent the largest proportion of macronutrients in quinoa seeds, accounting for 58–64% of the seed dry matter (DM), lower than that of wheat, corn, or rice (ranging from 78% to 81%) [30]. Polysaccharides are high-molecular-weight carbohydrates bound by glycosidic bonds with biological functions. Quinoa polysaccharides include starch polysaccharides and non-starch polysaccharides. In quinoa, starch serves as the most important carbohydrate (accounting for 60%), characterized by high solubility and digestibility with small granules of approximately 1.5 μm in diameter [1]. Quinoa has a higher content of amylopectin starch than amylose starch, and such a unique amylopectin structure gives quinoa starch special physicochemical properties such as low gelatinization temperatures and slow retrogradation [31]. Quinoa has low levels of free sugars, primarily sucrose, glucose, fructose, arabinose, and maltose, making it a low glycemic index grain particularly advantageous for individuals with diabetes.

Dietary fiber is the indigestible portion of plant-based foods that is digested and metabolized by microorganisms in the large intestine, mostly concentrated in the bran layers of the grains. According to the prebiotic property, dietary fiber can be classified as resistant oligosaccharides, non-starch polysaccharides, resistant starches, and associated substances [32]. Non-starch polysaccharides are the major components of quinoa fiber. The content of quinoa fiber ranges from 14% to 20%, higher than that of other cereals and more similar to fruits. Additionally, the content of insoluble fiber ranges from 10% to 14%, while soluble fiber ranges from 3.7% to 5.9% [12]. The composition of quinoa fiber was pectins (4.7%), hemicellulose (41%), lignins (1.7%), and cellulose (52%) [33]. The insoluble fiber in quinoa primarily comprises homogalacturonans interspersed with rhamnogalacturonan I units, which include galactans and arabinans as sidechains (55%), xyloglucans (30%) primarily with di- and trisaccharide sidechains, and cellulose. The soluble fiber mainly consists of arabinans and homogalacturonans [34]. It was reported that the soluble dietary fiber (SDF) of quinoa had higher hydroxyl radical scavenging ability compared to wheat SDF, with red quinoa SDF exhibiting superior functional characteristics such as higher thermal stability, gel-forming capacity, bile acid-binding capacity, and water-holding capacity than that of white and black varieties [35,36]. In addition to genetic factors, the composition of quinoa fiber is also influenced by processing methods and growing conditions [37].

### 2.3. Lipids

The content of lipids in quinoa ranges from 4.4% to 8.8%, surpassing traditional grains like corn, rice, and millet, making it a potential resource for oil extraction. The content of fatty acid (FA) in the embryo of quinoa is higher than that in the endosperm, pericarp, and seed coat. The average lipid level in quinoa is 5.3%, with 85% comprising FA. Approximately 11% of FA were saturated, with palmitic acid predominating [38]. The unsaturated FA of quinoa mainly includes oleic acid (19.7–29.5%), linoleic acid (49.0–56.4%), and α-linolenic acid (8.7–11.7%), similar to soybean composition [1,17,38]. Compared to light-colored varieties, dark-colored quinoa seeds had higher FA content, with unsaturated FA reaching 89.42% and an omega-6/omega-3 FA ratio of ca.6/1 [39]. The lipid profile of quinoa altered throughout the growth stages. With growth, α-linolenic acid was the predominant FA, ranging from 385 to 473 g/kg of total, whereas linoleic acid gradually decreased until the shoot stage then increased. Palmitic acid, oleic acid, and stearidonic acid showed consistent content during the process [40]. Moreover, processing methods and climate change also influence the FA content, composition, and proportions [41]. In one study, the content of polyunsaturated FA in quinoa irradiated by an electron beam increased by 3% at 1 kGy, and the value of omega-3/omega-6 (0.21) increased by 5% [42]. Quinoa lipids are known for their relative stability and resistance to oxidation, even after heating or exposure to oxygen. The high oxidative stability of quinoa lipids and seed oil may be attributed to the presence of vitamin E, especially γ-tocopherol, as natural antioxidants [39,43,44].

### 2.4. Vitamins and Minerals

Quinoa seeds contain bioavailable vitamins, including vitamin C, vitamin B6, vitamin B5, thiamine, and folate. Vitamin C is an essential nutrient with antioxidant capacity that must be acquired exogeneously from food. The vitamin C content in 100 g of quinoa leaves ranges from 70 to 230 mg, 40–52 mg for 4-day-old sprouts, 37–70 mg for 20-day-old seedlings, and 16 mg for seeds, which is undetectable in wheat, corn, or rice [45]. Additionally, 100 g quinoa contains high levels of vitamin B6 and folic acid, basically meeting the daily requirements of an adult. In particular, riboflavin in 100 g of quinoa meets 80% of children’s and 40% of adults’ needs [12]. Furthermore, quinoa is a good source of vitamin B2, fulfilling approximately 40% of the daily recommended intake for an adult [46]. Quinoa’s ash content (3.4%) is higher than that of rice (0.5%), wheat (1.8%), and most other grains. With regard to meeting the recommended daily intake of minerals, 100 g of quinoa is capable of fulfilling the daily needs of adults for magnesium, manganese, copper, and iron, while phosphorus and zinc provide 40–60% of the daily requirements [12]. Moreover, the mineral of quinoa was found in biologically appropriate forms for better absorption [16]. Importantly, during germination, the iron, calcium, and zinc contents of quinoa can be further enhanced, increasing by approximately 39.43%, 49.04%, and 20.25%, respectively [47]. Such a process is due to the germination activating enzymes within the seeds (α-amylase, proteases, pullulanase, phytase, and other glucosidases), which degrade antinutritional factors and break down complex macronutrients to release more minerals [48,49]. Additionally, antinutritional factors like phytic acid and tannin, which usually bind minerals and reduce their bioavailability, are significantly reduced during germination, thereby increasing mineral availability [50,51]. Finally, cell wall hydrolysis and new metabolic activities during germination also enhance mineral release or synthesis [52].

### 2.5. Polyphenols

Polyphenols are a diverse group of bioactive secondary metabolites produced by plants, characterized by the presence of one or more aromatic rings and hydroxyl groups. Polyphenols have two general classes: one is flavonoids, and the other is phenolic acids [53]. Among these, flavonoids are further divided into flavones, flavononse, flavonols, flavanols, isoflavones, anthocyanins, and phenolic acids are generally classified into hydroxybenzoic and hydroxycinnamic acids. Different colors and varieties of quinoa exhibit variations in the composition and content of polyphenols [54]. In red, white, and black quinoa, the total phenolic content (TPC) and total flavonoid content (TFC) ranged from 514.03 to 1409.54 mg gallic acid equivalent (GAE)/100 g and 177.49 to 407.75 mg rutin equivalent (RE)/100 g, respectively. Red quinoa showed the highest TPC and TFC with the best ABTS and DPPH radical scavenging activity. TPC in red and black quinoa are predominantly present in bound form, with protocatechuic acid being the most abundant phenolic acid but in free form in white quinoa [55]. At least 23 polyphenols were identified in quinoa, with the majority being phenolic acids, primarily including vanillic acid, ferulic acid, and their derivatives, as well as dominant flavonoids quercetin, kaempferol, and their glycosides. Notably, darker-colored quinoa seeds exhibited higher phenolic concentrations and antioxidant activity [56]. The anthocyanin content in quinoa was approximately 120.4 ± 7.2 mg cyanidine-3-glucoside equivalent (CGE) per 100 g DW, much higher than that of jasmine rice, Amaranthus hybridus, and soybean [57,58]. Generally, germination could further increase the TPC and antioxidant activity of quinoa [58,59]. Moreover, the environmental factors and variety of quinoa greatly influence the types and levels of polyphenols. Numerous studies have shown that fermentation of quinoa or its extracts with *Lactobacillus* or *Bifidobacterium* species significantly increased the TPC, bioavailability, and antioxidant capacity, suggesting a promising formulation for future health-promoting fermented foods [60,61,62,63]. Importantly, quinoa leaf extracts were another valuable source of polyphenolic compounds, with considerable amounts of ferulic, sinapinic, and gallic acids, as well as flavonoids like kaempferol, isorhamnetin, and rutin [64,65,66].

### 2.6. Saponins

Quinoa saponins, a typical antinutritional factor, serve a crucial function in shielding seeds from birds and insects due to their bitter taste and toxic effects. The basic structural formula of quinoa saponin is C_n_H_2n−8_O_10_ (n ≥ 5), which results from the glycosidic chain linking the carbohydrate chain to hydrophobic aglycones [67]. Quinoa-derived saponins are triterpene glycosides formed by the attachment of hydrophilic oligosaccharides at C-3 and C-28 positions of hydrophobic aglycone. Glucose, galactose, arabinose, glucuronic acid, and xylose are the common oligosaccharide sugars [68]. Saponins are the primary secondary metabolites in quinoa, mainly found in the outer layer of the seeds, with total content ranging from 3.81 to 27.1 mg/g [69]. Lim et al. found that quinoa seeds had significantly higher sapogenin content than stems, leaves, and roots, which were mainly composed of phytolaccagenic acid. Notably, quinoa roots had the highest amount of total saponin (13.39 g/100 g), followed by quinoa bran [70]. At least 40 saponin with eight different aglycones from quinoa have been isolated in the past 30 years, with the derived molecular entities essentially being phytolaccagenic, oleanolic, and serjanic acids; hederagenin; 3β,23,30 trihydroxy olean-12-en-28-oic acid; 3β-hydroxy-27-oxo-olean-12en-28-oic acid; and 3β,23,30 trihydroxy olean-12-en-28-oic acid [71,72]. The most common extraction methods for quinoa saponins include maceration, extraction by reflux, and Soxhlet, which rely on the solubility of solutes in solvents. Ethanol and methanol are considered optimal solvents for saponin extraction due to their high solubilizing capacity, with ultrasound and microwave assistance further enhancing extraction efficiency [73]. Key information on multiple saponins derived from quinoa is presented in Table 2.

### 2.7. Other Compounds

Quinoa contains minimal amounts of phytic acid, oxalates, betanin, phytosterols, and phytoecdysteroids. Phytic acid, a prevalent anti-nutrient compound in plant-derived foods, demonstrates robust chelating activity against minerals and other cationic molecules. Excessive consumption of phytic acid can reduce the absorption of divalent minerals, including iron, calcium, magnesium, and zinc. The phytic acid content in three colors of quinoa ranged from 1.03 to 1.22 g/100 g, which was decreased by enhanced phytase activity after germination [76]. Oxalate is another anti-nutrient factor that binds to essential minerals, thereby hindering nutrient availability. The total content of oxalates in quinoa seeds and roots ranges from 143 to 232 mg/100 g, while in leaves and stems, the content ranges from 874 to 1959 mg/100 g. Prolonged consumption of soluble oxalates reduces the bioavailability of minerals and trace elements, increasing the risk of developing calcium oxalate stones in the kidneys [77]. Betanin is a hydrophilic, water-soluble plant pigment with potent bioactive properties. Quinoa was found with higher amounts of betanin than other pseudocereals, approximately 3930 μg/g. Betacyanins, mainly betanin and isobetanin, were confirmed for the first time to be the pigments of red and black quinoa seeds instead of anthocyanins [56]. Plant phytosterols are a type of sterol that serve as crucial components of plant cell membranes and are known for anti-cholesterol functionality. It was reported that 100 g quinoa seeds contained 118 mg phytosterols, primarily consisting of β-sitosterol, campesterol, brassicasterol, and stigmasterol, surpassing barley, rye, millet, and corn in content [78]. Moreover, the level of phytosterols in quinoa seed oil reached 45.0 mg/g, with β-sitosterol being the most abundant (19.6–46.6 mg/g), followed by stigmasterol (8.7–20.1 mg/g) and campesterol (0.4–2.0 mg/g). However, the phytosterol content in quinoa flour was only 0.80 mg/g [78,79]. Quinoa also belongs to sources of phytoecdysteroids, with total content ranging from 138 to 570 μg/g, demonstrating potential pharmacological and metabolic bioactivities. At least 13 different phytoecdysteroids have been identified and isolated from quinoa seeds, with the most common being 20-hydroxyecdysone (20-HE), comprising 60–90% of the total phytoecdysteroids [80,81].

## 3. Bioactive Activities of Quinoa

**Figure 1:**
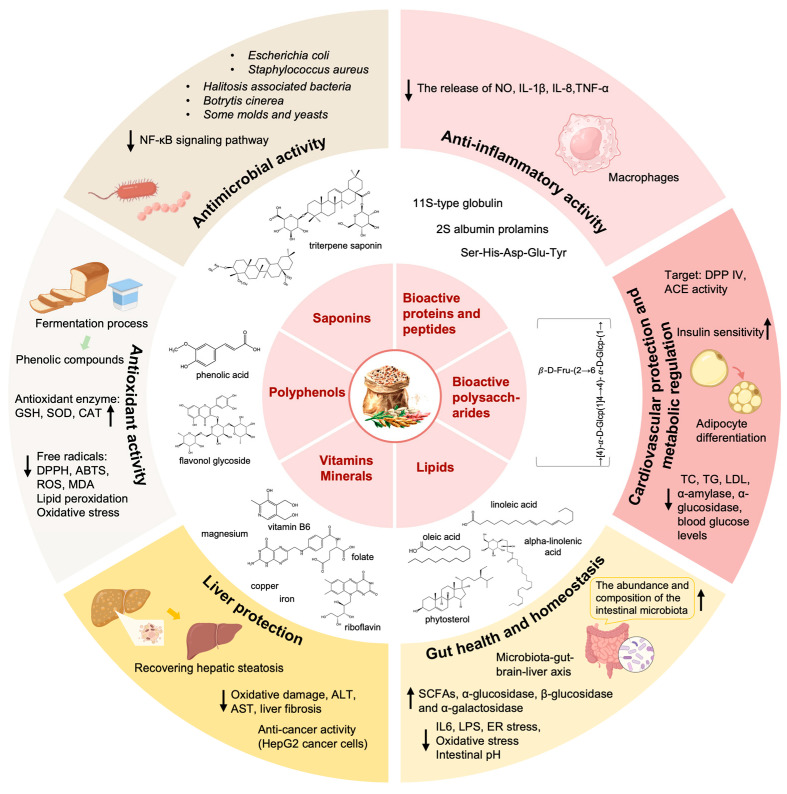
The bioactive activities of functional components derived from quinoa. Quinoa has various physiological functions. The bioactive proteins and peptides, polysaccharides, lipids, vitamins and minerals, polyphenols, and saponins in quinoa play different physiological functions, including but not limited to antioxidant activity, antimicrobial activity, anti-inflammatory activity, liver protection, CVD protection, metabolic regulation, and impact on gut health and homeostasis (↑: increase; ↓: decrease).

Quinoa possesses remarkable nutritional value, gluten-free characteristics, and therapeutic potential with possible nutraceutical benefits. Numerous studies have highlighted the multiple health-promoting benefits associated with quinoa consumption, attributed to its BAPs, polysaccharides, and unsaturated fatty acids, especially phytochemicals. Here, we comprehensively summarized the health benefits and molecular mechanisms of bioactive components derived from quinoa both in vivo and in vitro, providing theoretical support for developing quinoa as a novel functional food in the future (Figure 1).

### 3.1. Antioxidant Activity

In fact, all parts of the quinoa plant exhibit antioxidant activity, not just the seeds. A comprehensive comparison of total saponins, sapogenins, polyphenols, flavonoids, and antioxidant activity in different parts of quinoa plants was conducted and found that quinoa roots and sprouts had the highest content of flavonoids and phenolic acids, exerting stronger antioxidant activity than other parts [70]. In line with this, quinoa sprouts showed a higher level of total phenolic content and flavonoids and stronger antioxidant capacity compared to seed extracts [82]. Different quinoa varieties also exhibit varying levels of antioxidant capacity. In 25 yellow-seeded quinoa varieties, *Temuco* and *Rainbow* were identified as the most effective varieties in scavenging free radicals, while *Baer* and *Temuco* had the highest reducing power, indicating their capacity to donate electrons to ROS [83]. Many studies have unveiled that darker varieties of quinoa, like black quinoa, showed stronger antioxidant ability due to their higher level of total polyphenols and flavonoids. Moreover, free phenolics contributed the most (50–83%) to total phenolic content compared with conjugated or bound forms. Consistently, compared to white quinoa, extracts from black and red quinoa exhibited higher antioxidant activity, effectively reducing oxidative stress in HMEC-1 cells [84]. The latest research showed that the antioxidant activity and polyphenol content of quinoa was closely associated with the variety, as the darkest quinoa varieties, *Negra* and *Pasankalla*, exhibited the most favorable bioactive features regardless of their origin zone [85]. Interestingly, despite lower levels of total antioxidants compared to red quinoa, white quinoa’s richer content of bound phenolics demonstrated better DPPH and ABTS scavenging abilities, emphasizing the influence of polyphenol forms on antioxidant activity [55,56,74]. A recent study again confirmed that the darkest quinoa cultivars had the best functional properties and bioactive profile, underlining that genetic variability was more important than geographic factors [85]. Notably, the seed oil of black quinoa also exhibited the strongest antioxidant activity with the highest level of phytosterols, followed by red and white varieties [86].

The antioxidant capacity of quinoa is the integrated result of multiple bioactive components, especially BAPs, polysaccharides, unsaturated fatty acids, and polyphenolic compounds. In addition, vitamin C, vitamin E, and small amounts of carotenoids synergistically contribute to antioxidants. Daliri et al. reported that the DPPH radical scavenging capacity increased in the quinoa protein hydrolysate treated with trypsin digestion due to the expansion of protein molecules and availability of electron donor amino acids [20]. Further, quinoa peptides obtained by alkaline enzyme showed better antioxidant activity compared to trypsin hydrolysis [87]. Betanin-loaded nanocarriers based on 11S globulin from quinoa displayed stronger antioxidant efficacy than whole grains [88]. In detail, the albumin components of quinoa protein showed better antioxidant ability than globulins [89]. Lunasin, a representative BAP detected in quinoa, demonstrated strong ABTS radical scavenging activity (IC_50_ = 1.45 g/L) and oxygen radical scavenging activity [22]. Additionally, quinoa polysaccharide SQAP-2 exhibited outstanding capabilities for scavenging free radicals, including DPPH, ABTS, hydroxyl, superoxide radicals, and ferric iron reduction [90]. Another polysaccharide isolated from quinoa with high content and low molecular weight (8852 Da) purified by column chromatography exhibited a significant antioxidant effect against DPPH and ABTS [91]. Ethanol extraction of red quinoa bran yielded higher levels of rutin and total antioxidants. In vivo experiments indicated that feeding such extracts suppressed the expression of fatty acid synthesis enzyme acetyl-CoA carboxylase by increasing superoxide dismutase (SOD) activity while reducing alcohol-induced ALT and AST [92]. The hydrolysates from red quinoa improved renal oxidative stress in spontaneously hypertensive rats by reducing MDA levels and alleviating reduced SOD and glutathione (GSH) levels [93]. Al Qabba et al. found that ferulic acid and quercetin were the major phenolic acids and flavonoids in red quinoa and yellow quinoa sprouts, and more polyphenols were detected after germination. Importantly, quinoa sprout extracts markedly reduced MDA and efficiently enhanced the reduced GSH and SOD activities in oxidative stress-induced rats [94]. In the fructose-fed rat model, dietary supplementation of quinoa dramatically reduced oxidative stress levels in plasma, kidneys, liver, and lungs, manifested by a large decrease in blood MDA levels and lipid peroxidation biomarkers [95].

**Figure 2:**
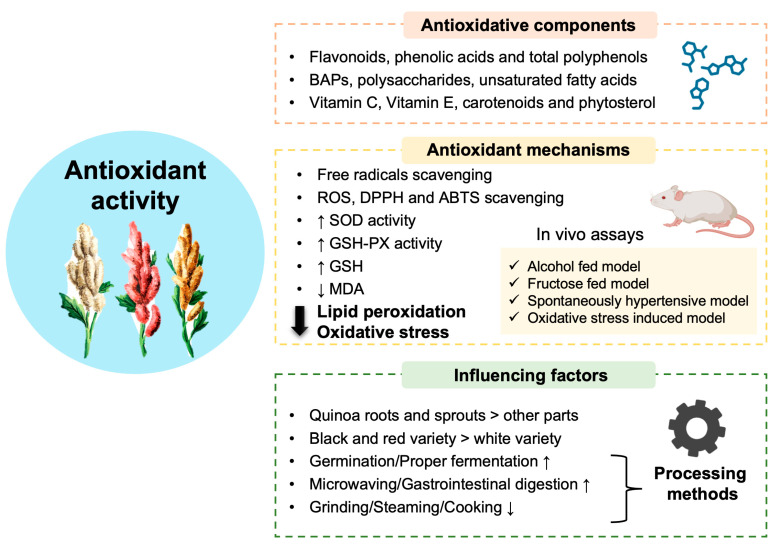
Quinoa exhibits excellent antioxidant capabilities. Quinoa is rich in various antioxidant components, most notably total polyphenols, flavonoids, and phenolic acids. Additionally, bioactive peptides (BAPs), polysaccharides, and unsaturated fatty acids also possess antioxidant functions. The vitamin family, including vitamin C (VC) and vitamin E (VE), along with small amounts of carotenoids and phytosterols, show antioxidant potential as well. These bioactive components exert their antioxidant effects by scavenging various free radicals like ROS and enhancing the activities of antioxidant-related enzymes such as superoxide dismutase (SOD) and glutathione peroxidase (GSH-PX) while reducing the levels of oxidative product malondialdehyde (MDA). In vivo assays induced by multiple models have shown that quinoa and its bioactive components effectively alleviate lipid peroxidation and systemic oxidative stress, consistent with the aforementioned indicators. The overall antioxidant activity of quinoa is significantly influenced by variety, plant parts, and different processing methods (↑: increase; ↓: decrease).

Different processing methods bring about changes in the antioxidant activity of quinoa. Grinding, steaming, and cooking led to reduced antioxidant activity due to the loss of total polyphenols and flavonoids by 31.5% and 41.4%, highlighting the importance of maintaining grain integrity [96]. Interestingly, microwaving released more polyphenols from black quinoa and enhanced the overall antioxidant activity [97]. Furthermore, gastrointestinal digestion of quinoa flavonoids led to a twofold increase in its antioxidant profile [98]. Importantly, germination and proper fermentation have been solidly confirmed to promote the antioxidant bioactivity of quinoa in multiple ways, especially by producing more BAPs and phytochemicals [62,99,100,101,102,103]. This section is illustrated in Figure 2.

### 3.2. Anti-Inflammatory Activity

Grains containing gluten, especially wheat, have been shown to possess a strong ability to activate TLR4 receptors, thereby increasing intestinal inflammation through the activation of intestinal and mesenteric lymph node myeloid cells [104]. Quinoa, in addition to being a gluten-free grain that does not trigger inflammatory responses, exhibits excellent anti-inflammatory activity both in vitro and in vivo due to its rich content of various active components such as BAPs, polyphenols, polysaccharides, and saponins, making it particularly beneficial for individuals with gluten intolerance and digestive diseases [105]. Herein, we mainly focused on the latest advancements in the anti-inflammatory field regarding quinoa and its functional components.

Saponins are the primary antinutritional factors in quinoa and represent strong anti-inflammatory properties. Four saponin components isolated from quinoa dose-dependently reduced the production of nitric oxide (NO), a typical inflammatory factor, and inhibited the release of inflammatory cytokines such as tumor necrosis factor-alpha (TNF-α) and interleukin-6 (IL-6) in lipopolysaccharide (LPS)-induced RAW264.7 cells [106]. It is well known that long-term consumption of high-fat diet (HFD), especially saturated and trans-fats, leads to inflammation and various chronic diseases. Total saponins extracted from quinoa bran significantly reduced IL-6 and LPS levels in HFD-induced obese rats while mitigating weight gain and visceral fat accumulation [107]. Lin et al. identified 11 saponins from quinoa bran, with the main components being hederagenin and oleanolic acid, which alleviated renal inflammation and kidney damage in hyperuricemic mice via inhibiting PI3K/Akt/NF-κB inflammatory signaling, restoring serum parameters to normal levels [108]. Franceschelli et al. identified 4′-geranyloxyferulic acid as the only phytochemical product found belonging to quinoa secondary metabolites, which effectively inhibited NOS, IL-6, and TNF-α release in HCT-116 cells [109]. A plethora of research has demonstrated that BAPs derived from quinoa possess potent anti-inflammatory effects, which are beneficial for vegetarians and individuals with celiac disease [110]. Capraro et al. described a novel bioactive peptide, chenopodin, derived from quinoa, which effectively inhibited NF-κB activation and IL-8 expression in Caco-2 cells under IL-1β-induced inflammation [91]. Further, feeding chenopodin reduced mice paw edema and neutrophil recruitment in carrageenan-induced inflammation in vivo [111]. Lunasin is a novel cancer-preventive peptide initially identified from soybean protein [112]. Purified lunasin isolated from various quinoa varieties significantly inhibited the production of NO, TNF-α, and IL-6 in LPS-induced RAW264.7 cells, with inhibition rates of 44.77%, 39.81%, and 33.50%, respectively [22]. However, another study pointed out that hydrolysates of quinoa protein prepared using papain, gastric protease, and pancreatin exhibited anti-inflammatory levels similar to those of total protein [113]. Capraro et al. found that none of the quinoa proteins or peptides induced inflammation in Caco-2 cells, but all protein components, especially albumin fraction, showed strong protection against inflammation induced by IL-1β [114]. Inflammatory bowel diseases (IBDs) are chronic inflammatory conditions that lead to the disruption of the colonic mucus barrier. A novel quinoa protein peptide, TPGAFF, was found to mitigate colitis in mice by restoring damaged mucus barrier and intestinal permeability, inhibiting activation of inflammatory signals and inflammatory cytokines. Interestingly, TPGAFF also affected the abundance and composition of inflammation-related gut microbiota [115]. The white albumin extracted from quinoa, with a molecular weight of less than 30 kDa, increased macrophage TLR4 expression in vitro and promoted CD68/CD206 ratio, demonstrating its impact on inflammation [116]. Consistently, quinoa ameliorated HFD-induced hepatocarcinoma severity by alleviating immune mediators in the intestinal under the pro-tumor microenvironment, including CD68/CD206 ratio [117]. In addition, four quinoa polysaccharides showed the activity to inhibit the level of TNF-α and IL-6 induced by LPS. Notably, polysaccharides extracted with water had better anti-inflammatory activity than that of alkali [118]. Another purified novel polysaccharide promoted the proliferation of RAW264.7 macrophages in vitro but inhibited the production of nitric oxide in a dose- and time-dependent manner [91]. Moreover, an in vivo assay showed that QPS1, a purified quinoa polysaccharide, improved the inflammatory response induced by cyclophosphamide via reducing the levels of IFN-γ, IL-6, and IFN-α in serum, as well as enhancing the phagocytic function of mononuclear macrophages [119].

Extracts from sprouted seeds of red and yellow quinoa, at a dose of 30 mg gallic acid equivalents (GAE) per kg, administered to CCL4-induced rat models significantly alleviated liver inflammation and restored serum indicators [94]. In an ethanol-induced gastric mucosal injury rat model, quinoa water extracts attenuated inflammation and repaired the damaged gastric mucosa via inhibiting Nrf2 and NF-κB signaling. Additionally, it also reduced the levels of inflammatory factors in H_2_O_2_-induced oxidative stress in GES-1 cells [120]. Red quinoa extracts, rich in antioxidants, counteracted oxidative stress and inflammation induced by CCl_4_ by inhibiting the secretion of TNF-α, IL-6, and transforming growth factor β1 (TGF-β1) in vivo, whose effectiveness surpassed the rutin [121]. Chronic metabolic disorders such as diabetes and obesity have been proven to be closely associated with inflammation, which is viewed as a central homeostatic mechanism [122]. Feeding db/db mice supplemented with quinoa remarkedly reduced protein carbonyls and IL-6, alleviating hepatic steatosis and total liver c accumulation that was presumed to be attributed to dietary fiber and phytochemicals [123]. In mice with HFD/streptozotocin-induced type 2 diabetes (T2DM), feeding quinoa yogurt effectively downregulated the expression of pro-inflammatory cytokines (TNF-α, IL-6, and IL-1β) while increasing the release of anti-inflammatory cytokine IL-10, alleviating systemic inflammation mediated by lipid dysregulation [124]. Apart from its active components, quinoa is promising to be utilized for anti-inflammatory applications for other drugs. Hydrolyzed quinoa protein as a carrier micelle stabilized anti-inflammatory drugs through the gastrointestinal tract and prolonged drug release. Animal experiments further confirmed that these micelles effectively accumulated in the inflamed colon region [125]. Collectively, the above findings suggest that quinoa may be used as a functional food component for the prevention and treatment of inflammation.

### 3.3. Antimicrobial Activity

The antimicrobial activity of quinoa is mainly due to its bioactive compounds like phenolics, saponins, and BAPs from seeds, leaves, roots, and inflorescences. Saponins, a class of secondary metabolites present in quinoa, were identified as major antimicrobial components. Alkali-treated quinoa saponin extracts exerted a noticeable inhibition on mycelial growth and spore germination of *Botrytis cinerea* due to increased affinity between saponin derivatives and sterols in the cell membrane [126]. Six saponins derived from quinoa husk strongly inhibited the growth of *Staphylococcus aureus* and *Staphylococcus epidermidis*, with MIC values of 0.0625 mg/mL and 0.125 mg/mL, achieved through inducing bacterial biofilm system collapse [127]. Sun et al. evaluated the inhibitory activity of quinoa saponins against several bacteria associated with halitosis and found that alkaline-converted quinoa saponins exhibited stronger inhibitory effects than resin-obtained quinoa saponin, particularly against *Fusobacterium nucleatum*, with a low MIC of 31.3 μg/mL [128]. Farajzadeh et al. evaluated the antibacterial activity of ethanol extracts from quinoa seeds of Giza1, Red Carina, and Sajama. The results indicate that Giza1 extract had the highest antibacterial effects. The MIC of Giza1 against *L. monocytogenes* and *E. coli* bacteria were 10 and 5 mg/mL, respectively [129]. The antimicrobial properties of quinoa’s abundant polyphenols are well-known, and fermentation further enhanced such activity by releasing more compounds [130]. Crude polysaccharide extracts of quinoa inhibited the growth of methicillin-resistant *Staphylococcus aureus* and *Escherichia coli* [131]. Moreover, the quinoa hydrolyzed protein peptides obtained using pepsin and alkali exhibited extremely high antibacterial activity against Gram-positive *Streptococcus pyogenes* and Gram-negative *Escherichia coli* [132]. Interestingly, the extract from quinoa inflorescences also contained various effective bioactive components that exhibited antibacterial effects, suggesting the potential optimization for utilizing different parts of the quinoa plant [133].

Microbial spoilage that occurs during food processing, storage, and transportation poses significant challenges to food safety, hygiene, and economic loss. Recently, the antibacterial and health-promoting activity of quinoa has led to its use as a natural food preservative. The active biofilm prepared from quinoa starch and gold nanoparticles exhibited strong antibacterial activity against multiple foodborne pathogens, with an inhibition rate of 99% against *Escherichia coli* and 98% against *Staphylococcus aureus* [134]. During a 12-day frozen period, the total counts of *Staphylococcus aureus*, molds, and yeast in hamburgers encapsulated with quinoa peptide liposomes were significantly reduced by over 50% compared to the control [135]. Another type of edible coating containing chitosan and quinoa protein greatly inhibited the growth of *Staphylococcus aureus* and *Salmonella species* [136]. Chitosan-nanoencapsulated extracts from red quinoa and ginseng have been shown to reduce the counts of various test bacteria (*Staphylococcus aureus*, *Bacillus subtilis*, *Salmonella*, *Escherichia coli*) [137]. Robledo et al. prepared nano-emulsions coated with quinoa and found it potently inhibited *Botrytis cinerea* growth in tomatoes after 7 days [138]. The antimicrobial properties of bioactive components within quinoa make it promising to be used as a novel packaging material for the maintenance of food safety and extension of shelf life.

### 3.4. Cardiovascular Protection and Metabolic Regulation

Excess body weight, hypertension, and dyslipidemia are regarded as the most potent established risk factors for CVD. Quinoa possesses a well-balanced amino acid profile and is abundant in unsaturated fatty acids, dietary fiber, micronutrients, and phytochemicals. These nutritional components collectively contribute to its potential to promote cardiovascular health and prevent CVD. The latest human intervention studies and animal experiments indicated that quinoa consumption partially alleviated metabolic stress and protected cardiovascular health. However, such effectiveness was found to be insufficient, and the molecular mechanisms behind it required further investigation [139]. A recent randomized parallel clinical trial involving 138 participants showed that postprandial blood sugar, total cholesterol (TC), triglycerides (TG), low-density lipoproteins (LDL), and body mass index were significantly lower in the quinoa group, particularly increasing high-density lipoproteins (HDL) [140]. A clinical study based on elderly prediabetic subjects revealed that an 8-week intake of quinoa lowered postprandial blood glucose levels, which was accompanied by reduced body weight [141]. Another randomized-controlled, double-blind crossover trial demonstrated that healthy older adults consuming 15 g quinoa cookies daily for 4 weeks experienced significant reductions in total and LDL cholesterol concentrations, weight, and BMI compared to the control. However, no significant changes were observed in TG, HDL, or PUFA [142]. The dietary intervention study based on 37 healthy overweight men showed that compared to refined wheat bread, daily consumption of bread containing 20 g quinoa flour demonstrated regulation of blood glucose levels but had minimal impact on other CVD risk biomarkers [143]. A prospective and double-blind study was conducted on 35 women with weight excess who consumed 25 g of quinoa flakes or corn flakes daily for four weeks. Surprisingly, the reduction of TC, LDL, and the increase in GSH occurred only in the quinoa group [144]. Furthermore, several meta-analyses based on randomized controlled trials have demonstrated that quantitatively consuming quinoa effectively intervened in blood glucose and lipid metabolism, particularly significantly reducing serum TG and TC levels [145,146,147].

Numerous in vivo experiments demonstrated quinoa’s activity in regulating lipid metabolism to reduce the risk of CVD. Consuming quinoa significantly reduced TC, LDL, and oxidized LDL levels in obese db/db mice, alleviating hepatic steatosis to levels similar to the control group [123]. In a Wistar rat model, feeding sprouted or fermented quinoa reduced food intake, blood glucose, and plasma lipid levels, as well as the accumulation of epididymal adipose tissue [148]. A recent study showed that quinoa reduced obesity, abnormal glucose, and lipid metabolism in a high-fat diet (HFD)-induced mouse model, possibly by modulating gut microbiota including *Bacteroidetes*, *Actinobacteria*, and *Desulfovibrio* [149]. Many studies have indicated a link between dysbiosis of gut microbiota and metabolic syndromes. Saponins from quinoa bran or BAPs from quinoa protein reduced lipid levels, blood glucose, body weight, and hepatic lipid accumulation in vivo via regulating the gut microbiota abundance and composition, which involved molecular mechanisms such as PPAR signaling and inflammatory indicators [107,150]. Supplementation with quinoa extract rich in 20-HE reduced fat tissue accumulation and insulin resistance in HFD-fed mice by reducing adipocyte size and fat storage [80]. Further, 20-HE globally increased energy expenditure and caused a shift in glucose metabolism toward oxidation to the detriment of lipogenesis. In addition, 20-HE also reduced the accumulation of AGE pigments, ROS, and fat deposits in *Caenorhabditis elegans* [151]. Additionally, a bioactive polysaccharide, SQWP-2, isolated and purified from quinoa greatly inhibited adipocyte differentiation by suppressing PPARγ, C/EBPα, C/EBPβ, C/EBPδ, SREBP1C, and AP2 in 3T3-L1 adipocytes, suggesting a direct biofunction on adipocytes [152,153]. Quinoa extracts had little inhibitory effect on human platelet aggregation, suggesting that quinoa’s cardiovascular regulatory function may be independent of such effect [154]. Moreover, soluble and insoluble dietary fiber extracted from quinoa bran also improved lipid metabolism in mice with antioxidant capacity [155].

Postprandial hyperglycemia has been recognized as an important risk factor for CVD and type 2 diabetes mellitus (T2DM). In preclinical models of Lepdb/+ pregnant mice, administration of quinoa protein hydrolysate alleviated glucose intolerance, improved liver insulin signaling, and several peptides exhibited anti-dipeptidyl peptidase-4 (DPP-IV) activity, a key enzyme involved in blood glucose control [156]. In Wistar rats, feeding quinoa for 15 weeks improved glucose intolerance, pancreatic β-cell dysfunction, insulin resistance, and lipid accumulation [157]. Another in vivo assay revealed that quinoa yogurt intake lowered fasting blood glucose levels while elevated hepatic glycogen content in T2DM mice, possibly via Akt/AMPK/PI3K signaling [124]. Based on bioinformatic analysis and computer-aided simulations, globulins in quinoa had the potential to inhibit DPP-IV and angiotensin-converting enzyme (ACE) [158]. Furthermore, four BAPs exhibited the highest DPP-IV inhibitory activity in Caco-2 cells, suggesting that quinoa protein could serve as a promising source of DPP-IV targeted peptides [28]. Moreover, polyphenols and polysaccharides extracted from sprouted quinoa yogurt upregulated GLP-1 release and showed anti-DPP-IV in NCI-H716 cells [159]. In line with this, the phenolic constituents from black quinoa alleviated insulin resistance in HepG2 cells via IRS1/PI3K/Akt/GLUTs signaling [160]. Feeding HFD-induced obese mice with quinoa extracts reduced body weight and fasting blood glucose levels while improving lipid profiles. In particular, the flavonoid rutin plays a key role in blood sugar regulation [161,162]. α-amylase and α-glucosidase are crucial enzymes in metabolizing dietary carbohydrates. The bound polyphenols in quinoa extract, composed of ferulic acid and its derivatives, significantly inhibited α-glucosidase activity in vitro, effectively delaying starch digestion. In vivo experiments showed that feeding ICR mice with 50 mg/kg BP reduced postprandial glucose increases, equivalent to 20 mg/kg of acarbose [163]. It was reported that five crude polysaccharides derived from quinoa exhibited inhibitory activity against α-amylase and α-glucosidase in vitro and that solid-state fermented with *Bifidobacterium* spp. further enhanced such bioactivity [164,165].

Hypertension is the leading cause of CVD, and ACE is an essential enzyme involved in the renin–angiotensin–aldosterone system (RAAS), which regulates blood pressure and electrolyte balance. ACE inhibitors are commonly prescribed medications for the management of hypertension and heart failure. Therefore, ACE inhibition is widely used as an in vitro index to evaluate anti-hypertensive properties. The latest research suggested that utilizing the high-pressure-assisted enzymatic hydrolysis method released the most potent ACE-inhibitory peptides from quinoa [166]. Orally administered hydrolysate of red quinoa (1000 mg/kg/day) for 8 weeks effectively alleviated hypertension indicators in rats without affecting body weight and food intake [93]. Four tripeptides derived from quinoa were identified through PeptideRanker as promising novel ACE-inhibitory bioactive peptides [158]. Consumption of cooked red or black quinoa altered the ACE condition and oxidative stress in hypertension-induced rats, attributed to the presence of phenolic compounds [167]. Quinoa active ingredients, particularly BAPs, have been shown to exert ACE inhibitory activity, which is affected by fermentation or hydrolysis. In contrast to non-fermented quinoa, *Lactobacillus plantarum* fermented quinoa exhibited increased ACE inhibition, indicating more release of active components in quinoa [164]. Germinated quinoa ferments inoculated with *Lactobacillus casei* yielded protein peptides LAHMIVAGA and VAHPVF, demonstrating notable inhibitory effects against α-glucosidase and ACE [168]. Chirinos et al. showed that quinoa protein hydrolysate inhibited ACE-1 activity by 89.2% without change after simulated gastrointestinal digestion in vitro [169]. A novel BAP extracted from quinoa bran albumin, RGQVIYVL, exhibited excellent ACE-suppressing activity (IC_50_ = 38.16 μM) in vitro and in vivo. Importantly, molecular docking simulations indicated the potential binding between RGQVIYVL and ACE active sites with high affinity [23]. Furthermore, another BAP isolated from quinoa bran globulin hydrolysate was found to bind non-competitively to the active site of ACE at Pro519 and Ser461 [170]. Feeding quinoa protein effectively reduced blood pressure in spontaneously hypertensive rats. Analysis of gastrointestinal contents and gut microbiota composition revealed promising precursor peptides [171,172]. Interestingly, incorporating quinoa into bread or plant-based milk has shown multifaceted metabolic regulation and CVD protective effects, again underlying the significance of quinoa as a functional food in preventing hypertension [173,174,175].

### 3.5. Protective Effects on Liver Function

The liver plays a critical role in numerous metabolic reactions, during which ROS, free radicals, and toxic metabolites are produced. Quinoa possesses antioxidant and anti-inflammatory properties, highlighting its potential as a hepatoprotective agent. Firstly, active components from quinoa reduce non-alcoholic fatty liver disease (NAFLD) through various molecular mechanisms such as antioxidation, anti-inflammation, and transcriptional regulation. Many in vivo studies have demonstrated that regular consumption of quinoa, quinoa extracts, or quinoa yogurt markedly alleviated multiple liver-related physiological indicators, including hepatic steatosis, lipid accumulation, hepatic inflammatory factors, liver function parameters (AST/ALT) and alcohol-induced liver damage, and systemic oxidative stress [92,176,177,178]. Interestingly, a study suggested that quinoa mediated the improvement of liver function through potential microbiota–gut–brain–liver interactions. Quinoa was found to modulate metabolism in the colon and liver by reducing ER stress and oxidative stress [149]. Quinoa and its extracts demonstrated preventive effects against hepatic steatosis, positively affecting the hepatic gene transcription program [123,155,179]. Indeed, quinoa was found to effectively upregulate lipid metabolism-related genes (Apoa5, Apoa4, Apoc2) and downregulate immune response-related genes (Irf5, Tlr6, Tlr10, Tlr11, Tlr12) to improve hepatic steatosis in rats [177]. Notably, the activation of ER stress markers eIF2α, GRP78, and CHOP mRNA expression in the liver is one of the potential mechanisms by which quinoa improves HFD-induced obesity in mice [180]. Moreover, the supplements of quinoa polysaccharides contributed to liver health by reversing HFD-induced hepatic steatosis in rats [160]. On the other hand, quinoa prevents the liver from various acute or chronic injuries and exerts a protective function. In the toxic model induced by CCl_4_, quinoa significantly alleviated liver toxicity by reducing oxidative stress and inflammation response, involving the molecular mechanisms of TGF-β1 signaling and ER stress pathway [94,121,177,181].

### 3.6. Regulative Effects on Gut Homeostasis and Microbiota

Observational findings achieved during the past two decades suggest that gut microbiota may contribute to the metabolic health of the human host and, when aberrant, to the pathogenesis of various common metabolic disorders, including obesity, T2DM, NAFLD, inflammatory bowel disease (IBD), cardio-metabolic diseases, and malnutrition [182]. Dietary interventions offer a direct or indirect means of altering gut microbiota to generate positive, beneficial effects on host health. Quinoa, with its abundance of nutrients and bioactive compounds, has been shown to exert positive effects on intestinal health and gut homeostasis. Numerous in vivo experiments have revealed that quinoa or its bioactive components alleviated intestinal disorder by modulating the composition and abundance of gut microbiota, aiding in the production of probiotics like *Bifidobacterium* spp. and *Lactobacillus-enterococcus* [60,183,184,185]. Herein, we mainly introduced the effects and mechanisms of quinoa bioactive components, including polysaccharides, saponins, and BAPs, on intestinal health and gut microbiota.

Polysaccharides are crucial for maintaining gut homeostasis and promoting the production of probiotics, particularly the production of short-chain fatty acids (SCFAs) such as butyrate, acetate, and propionate. Fecal fermentation of mixed grains (wheat combined with quinoa) yielded the highest level of propionate and butyrate compared with other formulations [186]. An in vitro fermentation with human fecal microbiota showed that quinoa substrates enhanced the growth of certain beneficial bacteria such as *Prevotella* and *Bacteroides*. Further, quinoa polysaccharides could be considered prebiotic due to their bioactivity to increase *Bifidobacterium* and *Collinsella* [187]. After 4 weeks of red quinoa polysaccharides intake, obese mice exhibited enhanced SCFAs and abundance in beneficial microbes *Akkermansia* [188]. Moreover, quinoa soluble fiber recovered the intestinal barrier and the abundance of *Lactobacillus* populations via increasing the number of goblet cells and Paneth cells [189]. Moreover, it was also found to reduce pathogenic varieties (*Bacteroidetes* and *Helicobacter pylori)* and enhance SCFAs, particularly acetate and butyrate, to improve ulcerative colitis [190]. Oral administration of quinoa polysaccharides increased the relative abundance and community structure of gut microbiota in rats, reducing the ratio of *Firmicutes* and *Bacteroidetes*, as well as decreasing the abundance of *Proteobacteria*, *Desulfovibrio,* and *Allobaculum,* which has been confirmed to be low in the gut of healthy human [191,192].

Saponins typically regulate intestinal function by exerting antioxidant, anti-inflammatory, and antimicrobial effects. Supplementation of quinoa saponins in HFD-induced obese rats resulted in an altered composition of gut microbiota compared to the control. Specifically, there was a decrease in the relative abundance of *Firmicutes phylum* and an increase in *Actinobacteria*, *Bacteroidetes*, *Bifidobacterium*, *Roseburia,* and *Coprococcus,* correlating with distinct metabolites [193]. Moreover, saponin-rich extracts of quinoa have shown evidence to be converted into sapogenin by human gut microbiota and exhibited a modulatory effect on the growth of gut microbes and α-diversity [194,195]. Compared to the HFD group, mice consuming saponin-containing quinoa upregulated the abundance of *Bacteroidetes*, *Actinobacteria*, and *Desulfovibrio* in the colon while reducing the abundance of *Blautia*. Additionally, quinoa increased the expression of TGR5 and GLP-1 in multiple tissues while downregulated the expression of TLR4 in the colon and liver, exerting the potential microbiota–gut–brain–liver interaction mechanisms [149].

**Figure 3:**
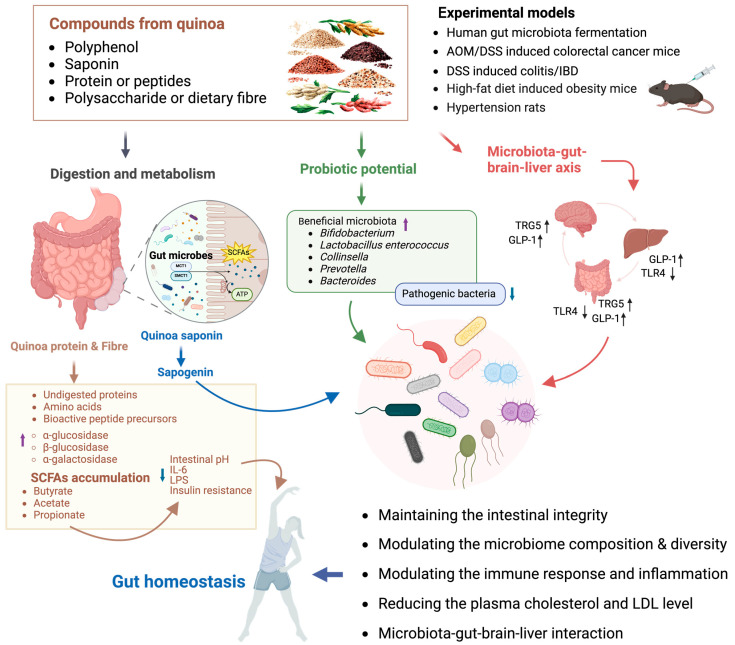
The potential mechanism of quinoa on human gut microbiota and intestinal homeostasis. Quinoa maintains intestinal homeostasis through multiple mechanisms. Its bioactive substances improve intestinal homeostasis by maintaining intestinal integrity, modulating the microbiome composition and diversity, modulating the immune response and inflammation, reducing the plasma cholesterol and LDL levels, and regulating the microbiota–gut–brain–liver interaction. Quinoa’s bioactive substances increase the activity of relevant enzymes in human body, breaking down complex carbohydrates into more easily absorbed forms, promoting the accumulation of short-chain fatty acids such as butyrate, stabilizing intestinal pH, regulating immunity and inflammation, and modulating insulin resistance, thereby regulating intestinal homeostasis. Additionally, quinoa’s bioactive substances show potential as probiotics, promoting the proliferation of beneficial bacteria, inhibiting the abundance of pathogenic bacteria, and maintaining intestinal homeostasis. Quinoa’s bioactive compounds also maintain intestinal homeostasis through the gut–brain–liver axis, specifically via promoting the increase in TRG5 and GLP-1, inhibiting TRL4, improving intestinal inflammation, and reducing liver fat accumulation (↑: increase; ↓: decrease; AOM/DSS: azoxymethane/dextran sodium sulfate; IBD: inflammatory bowel disease; SCFAs: short-chain fatty acids; MCT1: monocarboxylate transporter 1; SMCT1: sodium-coupled monocarboxylate transporter 1; TRG5: Takeda G protein-coupled receptor 5; GLP-1: glucagon-like peptide-1; TRL4: Toll-like receptor 4; IL-6: interleukin-6; LPS: lipopolysaccharide; LDL: low-density lipoprotein).

Quinoa protein, when digested in the hindgut, yielded undigested proteins and amino acids that can be used to produce SCFAs in the colon, lowering the intestinal pH and regulating microbiota growth in vivo [183]. Interestingly, the fecal microbiota in quinoa protein-treated spontaneously hypertensive rats shared more features in the composition of genera with non-hypertension rats than that of the captopril-treated group [171]. Multiple in vivo experiments have shown that supplementation with quinoa-derived BAPs restored intestinal barrier damage and inflammation induced by AOM/DSS or HFD by increasing SCFA levels and altering gut microbiota composition and abundance, particularly the ratio of probiotics to harmful bacteria, with involvement of molecular mechanisms PPAR-α/γ and TLR4/IκB-α/NF-κB signaling [150,196,197]. Moreover, the gastrointestinal process of quinoa protein provided further support for its active regulation of gut microbiota composition. In hypertensive rats consuming quinoa protein, there was a significant increase in the α-diversity of fecal microbiota, along with an enhanced abundance of probiotic bacteria *Turicibacter* and *Allobaculum* [171]. A novel quinoa peptide, TPGAFF, was identified and proven to alleviate colitis in vivo by reducing the composition of inflammatory-associated gut microbiota through NF-κB-TRPV1 signaling [115]. Several studies suggest that quinoa and its bioactive components have the potential to regulate the potential microbiota–gut–brain–liver axis to alleviate various metabolic disorders and intestinal mucosal damage [149,176]. The protective effects of quinoa on intestinal health can be attributed to its prebiotic properties, modulation of gut microbiota composition, and recovery of the intestinal barrier (Figure 3).

### 3.7. Anti-Cancer Effect

Cancer, a complex and devastating disease, is characterized by the uncontrolled growth and spread of abnormal cells. Quinoa is a highly promising edible and medicinal plant due to its diverse functional components. Here, we reviewed the inhibitory effects of quinoa and its bioactive anti-cancer components in vitro and in vivo, providing a theoretical basis for the functional value of quinoa as an adjunct dietary approach in cancer management. Initially, numerous studies found that extracts of quinoa could inhibit cancer cell viability. An earlier study demonstrated the efficacy of quinoa extracts in inhibiting the in vitro proliferation of human acute leukemia lymphocytes [198]. Next, people compared the anti-cancer activities of several pseudocereals and found quinoa extracts had the strongest cytotoxic activity against multiple human cervical cancer cell lines (C4-I, HTB-35, HTB-34) due to the high level of phenolic components and antioxidant activity [199]. Moreover, the ethanol extracts of quinoa inhibited the proliferation of colorectal cancer cell line (HCT-116) by reducing inflammation-related NO production and decreasing cellular arginine uptake [109]. Another study conducted by Stikić characterized the polyphenolic profiles of two quinoa varieties, *Puno* and *Titicaca*, and identified seven new phenolic and flavonoid compounds exerting anti-cancer activity against HCT-116 cells [200]. In A549 lung cancer cells, quinoa extracts at a concentration of 1.92 mg/mL demonstrated potent anti-cancer activity by upregulating BAX and downregulating Bcl2 levels to promote apoptosis [201]. Another study demonstrated the cytotoxicity of quinoa extracts against liver cancer cell line HepG2 in a dose- and time-dependent manner with IC_50_ of 14.6 μg/mL [202]. The extracts of black quinoa exhibited strong cytotoxicity against breast cancer cell line MCF-7, which is attributed to its high antioxidant content [203]. In summary, extracts from quinoa exhibit remarkable anti-cancer activity against various cancer cell lines, making it a promising source for cancer prevention as part of a novel healthy diet.

With the development of techniques for the identification and separation of bioactive ingredients, more and more studies are beginning to unveil the cytotoxic mechanisms of individual functional components isolated from quinoa against cancer cells. Here, we mainly focused on polyphenols, BAPs, polysaccharides, and saponins. It has been reported that quinoa contains at least 23 phenolic compounds, significantly higher than other grains [54]. Continuous research is revealing newly identified polyphenolic compounds in quinoa, with the highest level found in black varieties [204]. Overall, the anti-cancer effect of most phenolic compounds is attributed to their robust antioxidant activity, which disrupts the oxidative balance within cancer cells, activating signaling pathways such as oxidative stress, apoptosis, and autophagy, ultimately leading to cell death [205]. With a high level of antioxidant capacity, phenolic compounds from red and black quinoa demonstrated anti-tumor bioactivity via dramatically downregulating NO production and inhibiting the proliferation of MCF-7 cells [55]. Besides the seeds, quinoa leaves represent a valuable resource of polyphenolic compounds, which act synergistically in chemoprevention and anti-cancer therapy by modulating oxidative stress and ROS-dependent intracellular signaling pathways. The extract of quinoa leaves was found to contain a considerable amount of ferulic, sinapinic, and gallic acids; kaempferol; isorhamnetin; and rutin, demonstrating potent anti-proliferative, motility-inhibitory, and gap junction communication-suppressing effects against prostate cancer cells in vitro [64]. Moreover, metal-based nanoparticles with quinoa leaf extracts exhibited promising anti-cancer activity against HepG2 cells at very low concentrations [206]. Moreover, the lowest MCF-7 cell line viability percentage (13.92%) was observed in the leaf extracts of the black quinoa at 1000 mg/mL concentration [203]. Notably, compared to red and white varieties, the seed oil of black quinoa, with the highest content of phytosterols, had the best antioxidant and anti-proliferation effect on HCT-116 cells by inducing severe apoptosis [86]. In vivo, feeding breast cancer rats with nano-encapsulated quinoa seed oil inhibited tumor proliferation, activated cell apoptosis, and suppressed the expression of TNF-α, MYC, and PIK3CA, with no hepatorenal toxicity observed [207].

BAPs of quinoa have gained popularity among researchers as a potential source of anti-cancer inhibitors in recent years. After in vitro gastrointestinal digestion, the portion of quinoa peptides with molecular weights >5 kDa exhibited potent anti-cancer activity against various cancer cell lines, including Caco-2, HT-29, and HCT-116 cells, suggesting its role in reducing gene mutations and chromosomal rearrangements [26]. Lunasin is a novel cancer-preventive peptide that was first isolated and purified from soybean protein. For the first time, Ren et al. detected the presence of lunasin in 15 quinoa samples and found it inhibited the production of ROS, NO, TNF-α and IL-6 in vitro, exhibiting antioxidant, anti-inflammatory, and potential anti-tumor activity [22]. Interestingly, a study found that compared to using olive leaf polyphenol extract (QLE) alone, the complex of digested quinoa peptides and QLE exhibited stronger inhibition of cell viability in MKN-45 human gastric cancer cells [208]. Fan et al. further identified several novel BAPs with highly anti-cancer bioactivity from quinoa protein digestion, which inhibited the proliferation of colon cancer Caco-2 cells by suppressing the expression of oncogenic genes NF-κB, IL-6, and Bcl-2 [209]. In an in vivo assay, supplementation with quinoa protein and its hydrolysate alleviated the progression of AOM/DSS-induced colon cancer by reversing the reduced SCFAs and dysregulated gut microbiota in colon tissue [196]. A recent study conducted by Galindo Luján utilized label-free mass spectrometry-based shotgun proteomics to obtain the proteome map of 1211 identified quinoa proteins, which was employed to identify quinoa proteins with potential immunonutritional bioactivities, including those associated with cancer [210].

Previous research has uncovered the anti-cancer efficacy of various plant-based polysaccharides. Two crude polysaccharide extracts of quinoa (Q-40 and Q-60) showed potent antioxidant abilities and inhibited the proliferation of HepG2 and MDA-MB-231 cell lines at very low concentrations [131]. Another purified quinoa polysaccharide constituted by galacturonic acid and glucose displayed cytotoxicity against liver cancer SMMC-7721 and breast cancer MCF-7 cells without inhibition on normal cells [91]. The terpenoids derived from quinoa bran (TQB) significantly promoted the death of colorectal cancer cell line DLD-1 and HCT-8, with IC_50_ values of 0.42 ± 0.02 and 0.54 ± 0.05 mg/mL, which was also validated in vivo. Mechanistically, TQB upregulated the level of pro-apoptotic effectors Caspase-3 and Bax while decreasing the anti-apoptotic protein Bcl-2 and mitochondrial membrane potential [211]. Further, three saponin components, namely ursolic acid, oleanolic acid, and hederagenin derived from quinoa, inhibited the growth of A549, SH-SY5Y, HepG2, and HeLa cells in vitro, promoting apoptosis via Caspase-3 and cleaved-PARP [212]. Ribosome-inactivating proteins (RIPs) are present in several edible plants, and numerous studies have highlighted their potential in anti-cancer therapy. Quinoin, a new RIP isolated from quinoa, strongly inhibited the growth of glioblastoma cells and exhibited synergistic sensitization with temozolomide [213].

**Figure 4:**
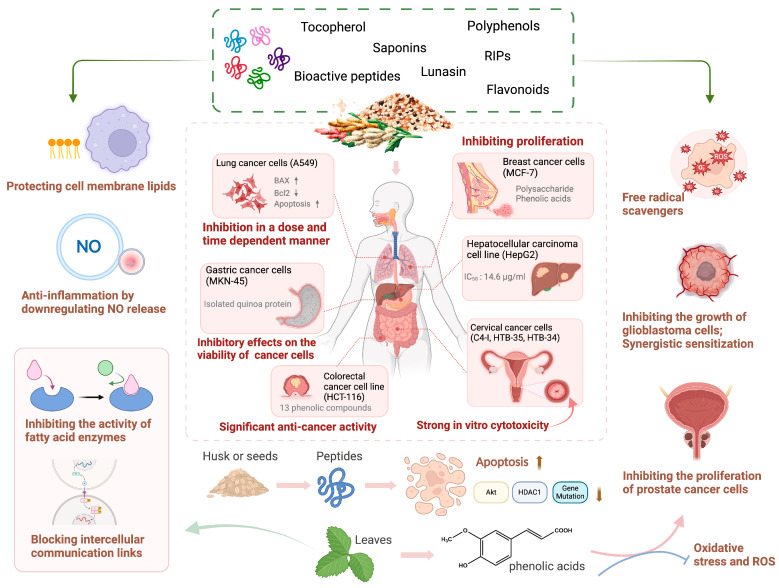
The inhibitory effect of bioactive components derived from quinoa on various cancer cells. Quinoa exerts anti-cancer effect via various mechanisms. Quinoa-derived bioactive components have strong inhibitory effects on the proliferation of multiple cancer cells, including lung cancer cells (A549), gastric cancer cells (MKN-45), breast cancer cells (MCF-7), liver cancer cells (HepG2), cervical cancer cells (C4-1, HTB-34, HTB-35), and colorectal cancer cells (HCT-116). Such inhibitory effect is primarily achieved by promoting cell apoptosis, inhibiting cell division, downregulating NO release to exert anti-inflammatory effects, scavenging free radicals, and inhibiting signaling pathways such as Akt and HDAC1. Additionally, the active components of quinoa promote cancer cell death through synergistic effects with clinical drugs (↑: increase; ↓: decrease; NO: nitric oxide; ROS: reactive oxygen species; O2−: superoxide anion; BAX: bcl-2-associated X protein; Bcl2: B-cell lymphoma 2; Akt: protein kinase B; HDAC1: histone deacetylase 1).

Collectively, the above studies demonstrate that quinoa and its bioactive components possess strong inhibitory effects on multiple cancer cells, making it a promising natural product with great potential for cancer treatment. However, in vivo studies and human clinical trials are limited, and the precise target of quinoa needs to be explored and verified in the future (Figure 4) (Table 3).

## 4. Conclusions and Future Perspectives

**Figure 5:**
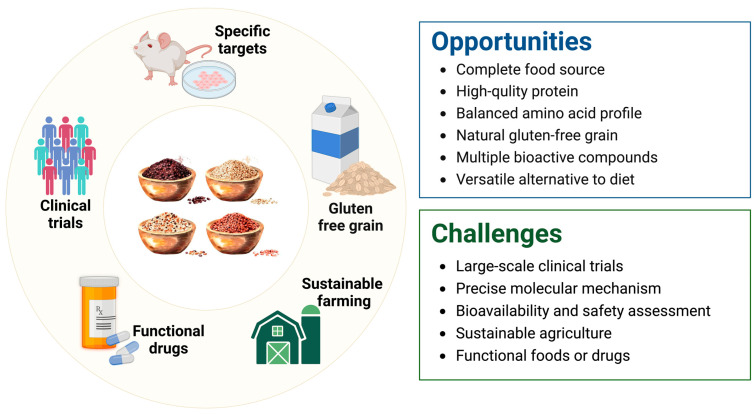
The challenges and opportunities of quinoa in future applications.

Quinoa has gained popular attention not only for its rich and balanced nutritional profile but also for its various bioactive compounds, such as polysaccharides, BAPs, polyphenols, and saponins. Many in vitro and in vivo experiments have confirmed that quinoa and its bioactive components have preventative or therapeutic effects on multiple human diseases, including CVD, metabolic syndromes, gastrointestinal disorders, and cancer. As a result, quinoa holds immense potential for development into functional foods with many health-promoting benefits. Despite the positive functional value of quinoa, there remains a significant gap in research regarding its specific targets in cell experiments, particularly in human clinical trials. Although there are a few results from dietary intervention studies focusing on obesity and hyperglycemia, research in areas of cancer prevention and gut regulation is virtually absent. Therefore, further well-designed clinical trials should be conducted to confirm the health benefits of quinoa and its derivatives. Furthermore, the deep exploration of protein targets interacting with quinoa bioactive components within cells using advanced technologies such as human proteome microarray technology, computer-aided drug design system, and DARTS assays is quite necessary for elucidating mechanisms and advancing new drug development (Figure 5).

A variety of in vivo experiments have demonstrated the regulatory effects of functional quinoa products, such as yogurt, especially those containing sprouted quinoa, on NAFLD, hypercholesterolemia, intestinal inflammation, and gut microbiota abundance. However, there is limited research on population trials and the impact of processing methods on the bioactive compounds based on these functional foods, highlighting the urgent need for exploration. In addition, further testing and optimization are required to increase the content and stability of bioactive components in quinoa functional foods.

## Figures and Tables

**Table 1:** Typical peptides isolated from quinoa samples.

Bioactive Compound	Analysis Method	Typical Substances	Reference
Lunasin	UPLC-ESI-MS	2.84 μg/g DW	[22]
Protein hydrolysate	LC-MS	MMFPH, FDLSHGS, LDKFLST, KTLPAVF, TKLPAVF and PPFLQVVPEV	[24]
Protein hydrolysate	ESI-Q-TOF-MS/MS	FHPFPR, NWFPLPR, PNFHPFPR, NIFRPF, SLPNFHPFPR, INNIFRPF, SVENWFPLPR, NSLSLPNFHPFPR, NSWGPNWGDHG, HYNPYFPGGA	[25]
Protein concentrate	LC-MS/MS	LWREGM, DKDYPK, DVYSPEAG, IFQEYI, RELGEWGI	[26]
Protein concentrate	RP-HPLC-MS/MS	peptide fragments included IQAEGGLT, DKDYPK, GEHGSDGNV	[27]
Protein-derived peptides	UPLC-MS/MS	IPI, IPV, VAYPL, IPIN, IPIIN, APW, IPAV, VPF, IPF, IPNPI, WPI, LPY, FAYP, FAMP, LALP, MPAGV, FAMPA, LAFPH, LPW, IPR	[28]
Protein-derived peptides	LCMS Q-TOF	FFE, DFTF, DFLM, ML, CDCP, CYTF, FSAGGLP	[29]
Protein-derived peptides	LC-MS/MS	RGQVIYVL, ASPKPSSA, QFLLAGR	[23]

**Table 2:** Contents of saponin in various quinoa samples.

Bioactive Compound	Analysis Method	Samples	Contents	Reference
Total saponin	Spectrophotometry	Available sample (Hongcheon, Republic of Korea)	13.39 g/100 g	[70]
	Spectrophotometry	4 available quinoa varieties (Shanxi, China) and 3 available quinoa varieties (Peru)	7.51–12.12 mg OAE/g DW	[74]
	Spectrophotometry	28 quinoa varieties (Brigham Young University, Washington State University, and other sources)	3.81–27.1 mg/g	[69]
	Spectrophotometry	Commercially available sample (Moreno Valley, CA, USA)	97.03 ± 1.53 mg OAE/100 g	[75]
Oleanolic acid	GC-MS/MS	Available sample (Hongcheon, Republic of Korea)	0.301 mg/g	[70]
	GC-MS/MS	28 quinoa varieties (Brigham Young University, Washington State University, and other sources)	0.896 mg/g DW	[69]
Hederagenin	GC-MS/MS	Available sample (Hongcheon, Republic of Korea)	0.300 mg/g	[70]
	GC-MS/MS	28 quinoa varieties (Brigham Young University, Washington State University, and other sources)	1.841 mg/g DW	[69]
Phytolaccagenic acid	GC-MS/MS	Available sample (Hongcheon, Republic of Korea)	1.650 mg/g	[70]
	GC-MS/MS	28 quinoa varieties (Brigham Young University, Washington State University, and other sources)	7.818 mg/g DW	[69]
Sejanic acid	GC-MS/MS	28 quinoa varieties (Brigham Young University, Washington State University, and other sources)	0.459 mg/g DW	[69]

**Table 3:** The effects of bioactive components derived from quinoa on multiple cancer cells.

Compounds	Cell Line	Cancer Type	Treatments/Conditions	Functional Activity	References
Purified quinoa polysaccharides Quinoa seed extract	MCF-7	Breast Cancer	IC_50_ for 24 h and 48 h were 83.48 μg/mL and 64.67 μg/mL	Strong inhibition of MCF-7 but no inhibition on normal cells	[91]
	SMMC-7721	Liver Cancer	IC_50_ for 24 h and 48 h were 121.4 μg/mL and 53.4 μg/mL	Strong inhibition of SMMC 7721 but no inhibition on normal cells	[91]
	HCT-116	Colorectal Cancer	The 48 h IC_50_ of two varieties of quinoa (*Ponu* and *Titicaca*) were 110.68 μg/mL and 239.47 µg/mL, respectively	Significant inhibition of proliferation	[200]
	A549	Lung Cancer	Dose-dependent 1.60 mg/mL and 1.92 mg/mL	Inhibiting cell viability; inducing strong apoptosis (increased BAX and decreased Bcl2 levels).	[201]
Quinoa leaf extract	MAT-LyLu	Prostate Cancer	Dose-dependent 0.186–1.86 mg/mL	Blocking intercellular communication connections and inhibiting cancer cell proliferation	[64]
	AT-2	Prostate Cancer	Dose-dependent 0.186–1.86 mg/mL	Blocking intercellular communication connections and inhibiting cancer cell proliferation	[64]
Peptides derived from quinoa	HCT-116	Colorectal Cancer	>5 kDa peptides showed IC_50_ = 0.176 ± 0.000 mg protein/mL, <5 kDa peptides showed IC_50_ = 0.928 ± 0.012 mg protein/mL	Inhibition of cell proliferation	[26]
	HT-29	Colorectal Cancer	>5 kDa peptides showed IC_50_ = 0.085 ± 0.003 mg protein/mL, <5 kDa peptides showed IC_50_ = 0.781 ± 0.009 mg protein/mL	Inhibition of cell proliferation	[26]
	Caco-2	Colorectal Cancer	>5 kDa peptides showed IC_50_ = 0.239 ± 0.001 mg protein/mL, <5 kDa peptides showed IC_50_ = 0.676 ± 0.007 mg protein/mL	Inhibition of cell proliferation	[26]
Novel bioactive peptides FHPFPR, NWFPLPR, and HYNPYFPG obtained from quinoa protein digestion production (<5 kDa)	Caco-2	Colorectal Cancer	IC_50_ was 0.87 g/L, 1.27 g/L and 1.85 g/L, respectively	Significant inhibition of Caco-2 cell proliferation by inhibiting histone deacetylase 1 (HDAC1) activity and regulating oncogenic gene expression	[209]
Saponin-rich quinoa extract (QE) and its hydrolyzed extract as sapogenin-rich extracts (HQE)	DLD-1	Colorectal Cancer	48 h IC_50_ values close to 100 μg/mL	-	[214]
	SW620	Colorectal Cancer	48 h IC_50_ values close to 100 μg/mL	-	[214]
Quinoa ferments	Caco-2	Colorectal Cancer	-	Cytotoxic effect	[164]
	MCF-7	Breast Cancer	-	Cytotoxic effect	[164]
Quinoa extract	C4-I	Cervical Cancer	1 mg/mL	Cytotoxic effect; a decrease in death cells by 28%	[199]
	HTB-35	Cervical Cancer	1 mg/mL	Cytotoxic effect; a decrease in death cells by 33%	[199]
	HTB-34	Cervical Cancer	1 mg/mL	Cytotoxic effect; a decrease in death cells by 45%	[199]
Complexation of quinoa protein isolate (QPI) combined with different concentrations of olive leaf polyphenol extracts (OLE)	MKN-45	Gastric Cancer	IC_50_ of QPI was 2.89 µg/mL	Inhibition of cell viability	[208]
Phenolic compounds of white, red, and black quinoa	MCF-7	Breast Cancer	For free fractions, IC_50_ values of white, red, and black quinoa were 2.20 mg/mL, 1.86 mg/mL, and 1.3 mg/mL, respectively; for bound fractions, IC_50_ values were 2.34 mg/mL, 1.75 mg/mL, and 1.61 mg/mL, respectively	Concentration-dependent inhibition of the proliferation	[55]
Seed oil of white, red, and black quinoa (WSO, RSO, BSO)	HCT-116	Colorectal Cancer	IC_50_ of BSO WSO and RSO were 281.9, 381.3, and 647.4 µg/mL, respectively	Apoptotic rates in HCT-116 cells treated with BSO at 125 and 250 μg/mL for 36 h were 10.4% and 21%	[86]
Water soluble terpenoid isolated from quinoa bran (QBT)	HCT-8	Colorectal Cancer	IC_50_ of QBT was 0.54 mg/mL	Promoting apoptosis; inducing caspase-related pathways and mitochondrial membrane potential changes	[211]
	DLD-1	Colorectal Cancer	IC_50_ of QBT was 0.42 mg/mL	Concentration dependence	[211]
Quinoa extract and its component 4′-geranyloxyferulic acid (GOFA)	HCT-116	Colorectal Cancer	-	GOFA interfered with the expression of the CAT-2B transporter, decreased the uptake of L-arginine of HCT-116	[109]
Quinoin (novel type 1 RIP derived from quinoa seeds)	U87Mg NULU ZAR	Glioblastoma	2.5 and 5.0 nM	Cytotoxicity effect; exhibiting synergistic sensitization with drug temozolomide	[213]
